# An interdisciplinary team’s experiences of a university-led community engagement intervention in the Tshwane district

**DOI:** 10.4102/hsag.v30i0.2831

**Published:** 2025-04-03

**Authors:** Hafsa Essop, Eugene Machimana, Mable Kekana

**Affiliations:** 1 Department of Radiography, Faculty of Health Care Sciences, University of Pretoria, Pretoria, South Africa; 2 Department for Education Innovation, Unit for Community Engagement, University of Pretoria, Pretoria, South Africa

**Keywords:** abandoned babies, community engagement, higher education, University-led, interdisciplinary team

## Abstract

**Background:**

The Bags of Hope (BoH) intervention is an interdisciplinary community engagement intervention bringing health professionals and students together towards uplifting the community. Through the project, abandoned newborn babies and mothers in need are provided with care packages. The project entails collecting, creating and distributing care packages within training hospitals.

**Aim:**

The aim of the study was to explore the experiences of the interdisciplinary team as to how the BoH intervention impacted their working relationships and care towards the beneficiaries.

**Setting:**

The study was conducted in three training hospitals within the Tshwane district of South Africa and University of Pretoria.

**Methods:**

This was a qualitative, exploratory study. The study population included radiography students (three), qualified radiographers (three), nurses (two) and social workers (two) involved in the BoH intervention. The data were analysed using thematic analysis.

**Results:**

From the data analysis, four themes were generated: (1) The BoH intervention as a potential abandonment prevention strategy for hospital born infants, (2) psychological impact of the BoH intervention on the interdisciplinary team, (3) strengthening of interdisciplinary collaboration and service delivery through the BoH intervention, (4) sustainability strategies for the BoH intervention.

**Conclusion:**

The BoH intervention provided immediate relief to abandoned babies and mothers in need. The interdisciplinary team was emotionally impacted, experiencing a ‘warm glow’ effect. To sustain the intervention, increased public awareness, along with ensuring all healthcare professionals are well informed, is crucial for its long-term success.

**Contribution:**

The research highlights the value that interdisciplinary collaborations can have towards uplifting communities, improving working relations and bridging resources gaps.

## Introduction

Abandonment of neonates can be categorised as ‘abandoned infants’ and ‘discarded infants’ (Mueller & Sherr 2009). The first refers to babies who are left in hospitals whereby the mother absconds shortly after giving birth (Mueller & Sherr 2009). Discarded infants are babies who are discarded in a public place without care (Mueller & Sherr 2009). They, if found in time, are often brought to hospital for further care until a place of safety is arranged (Guha et al. 2019). This purposeful neglect can therefore lead to neonaticide, which is the act of killing a baby in the first 24 h of its life (Friedman & Resnick 2009). However, babies who are abandoned in public areas also suggest that the mother may have wanted the baby to live, even though the baby is unwanted (Güneş, Şengül & Erdem 2023).

In Italy, approximately 3000 babies are abandoned every year (Sherr, Roberts & Croome 2018), while in the United Kingdom, around 400 babies are abandoned in hospitals annually (Ferrara et al. 2013). Several case studies of child abandonment and neonaticide have also been documented in Denmark and Hong Kong (Gheorghe et al. 2011; Lii 2006). In 2015, child abandonment in South Africa was on the rise with an estimated 3500 abandoned babies a year, aged from birth to 2 years (Baloyi 2015). Causes of abandonment often include the baby’s health condition, maternal conditions and inadequate financial support to care for the baby (Abukari & Schmollgruber 2024). In 2020, the coronavirus disease 2019 (COVID-19) pandemic further complicated these factors, with job losses and social isolation (Health 2020). In 2020, the Gauteng Department of Health raised concerns over the increase of abandonment, with 118 abandoned babies in hospitals across Gauteng within 8 months, from the start of January to August (Health 2020). The Tshwane district also experienced this crisis with the media reporting four babies being abandoned in the month of July 2021 (Tlhabye 2021). In 2024, media house Herald (Caxton Network News 2024) continues to report on the surge of abandoned babies in the Western Cape, reflecting on 2023 statistics with 88 reported cases of abandoned infants, with 59 of these babies found dead (Caxton Network News 2024).

In response to dire circumstances, countries such as Germany, Poland, the Czech Republic, India and South Africa have since adopted baby hatches, to safely leave unwanted babies to caregivers (Okawa et al. 2009). The intervention was developed to prevent ongoing neonaticide, as well as to restore dignity and humanness to the abandoned child (Okawa et al. 2009). In Ghana, perceived interventions for abandoned babies include staff contributions to support care, donations from individuals and institutions, and the strengthening of the social welfare department (Abukari & Schmollgruber 2024). In the United Nations Sustainable Development Goals (SDGs) report for 2024, priority is placed on the good health and well-being of individuals through its SDG, particularly vulnerable women and children. The United Nations further places importance on the value of partnership and collaboration (SDG 17) in order to assist in achieving goals. The study therefore aligns well with SDG 17, whereby collaboration between higher education institutions and clinical training hospitals facilitated the provision of care packs to abandoned babies and mothers in need within these hospitals. This further addresses SDG 3, whereby vulnerable mothers and infants are provided with essentials, such as toiletries and clothes to provide short-term relief, ensuring well-being in the first few critical days of life to the infant.

This response aligns well with the concept of Ubuntu, which is an African philosophy based on integrating components of humanity and interconnectedness, whereby one sees themselves through others (Chigangaidze, Matanga & Katsuro 2022). Principles of Ubuntu include justice, caring, collectiveness and responsibility towards ensuring the well-being of individuals in a community (Mayaka & Truell 2021).

Over the past two decades, the role of higher education in promoting community engagement has been a significant focus in academic discussions, as well as within the public and private sectors. Meanwhile, civil society has increasingly embraced the discourse of community engagement (Abdi, Shultz & Pillay 2015). Community engagement is prominent in the United States and has also been adopted in Australia and South Africa (Bowen 2013; Boyer 1996; Bringle & Hatcher 2009). The South African Higher Education Quality Committee (HEQC) defines Higher Education Community Engagement (HE-CE) as the ‘combination and integration of teaching and learning, (e.g. [academic] service-learning), professional community service by academic staff and participatory action research applied simultaneously to identified community development priorities’ (Bender et al. 2006). The aforementioned definition acknowledges that HE-CE should be for mutual benefit and inclusive partnership between the HE and community partners (Burnett, Hamel & Long 2004). In addition, service and learning are equally important in HE-CE. Community engagement, scholarship of teaching and learning (SoTL) and research can be used as vehicles to respond to local, regional and global injustices and inequalities (Department of Education 1997).

Tshwane district is the precinct for clinical training in all Health Science programmes at the University of Pretoria. The University of Pretoria, Bags of Hope (BoH) intervention was started by the Department of Radiography in response to the dire situations facing abandoned babies and later on even mothers in need. Radiographers and students were often called to perform mobile x-rays on abandoned babies, which initiated their response towards assisting these vulnerable groups through a community engagement structure, which is well supported by higher education. The intervention included an ongoing partnership with training hospitals to provide care packages to offer temporary support to these babies and restore their dignity. The care packages included clothing, nappies, towels, blankets and toiletries such as soap and cream.

The intervention was conducted within three phases in collaboration with various stakeholders namely: (1) call for donations by university lecturer (intervention manager) and students, (2) creating care packages from donations (students) and (3) identification of recipient and distribution (social worker, nurse, qualified radiographer and student radiographers). Student radiographers who train in these hospitals engage with abandoned babies and the nursing team during radiographic procedures. Student radiographers do not directly engage with social workers; however, because of the sensitive nature of dealing with abandoned babies and mothers in need, it was important to include the social workers in the project to be able to identity appropriate recipients of the care packages. When the recipient was identified, the nurse or social worker would alert the radiography department, who would then take a care package with the student to the baby. This was the start of the interdisciplinary collaboration underpinning the BoH intervention.

This is an example of a university-led intervention aimed to uplift society. The intervention, however, is a new initiative and therefore needs to be assessed through the lens of the interdisciplinary team who are driving the intervention to ensure its sustainability.

In the current study, an interdisciplinary approach was undertaken. Interdisciplinary collaboration involves different disciplines coming together for a common cause (Aboelela et al. 2006). It is often utilised when there are limitations recognised within individual disciplines when solving complex healthcare-related challenges (Aboelela et al. 2006). In this context, the radiography department is limited to diagnostic imaging. It was therefore imperative that the radiographers engage with nurses and social workers who deal directly with the babies, for distribution of the care packages to be undertaken in a safe and structured manner. Velempini (2020) further suggests that the effectiveness of community outreach implementation should be evaluated to identify areas whereby the programme can be improved upon (Velempini 2020). The purpose of this research was therefore to explore the experiences of the interdisciplinary team involved in the BoH to understand and describe how it was received by all stakeholders.

## Research methods and design

The study adopted a qualitative research design with a descriptive exploratory research approach. This was deemed an appropriate design as the study population was a small cohort of participants. The study aimed to gain an in-depth exploration of the interdisciplinary team’s experiences, namely nurses, social workers, radiographers and student radiographers, as well as their recommendations, which could be transferred to other similar settings.

### Study setting

The study was conducted at three training hospitals in the Tshwane district.

### Population and sampling

The total study population of the BoH interdisciplinary team included 23 members, consisting of nurses (*n* = 3), social workers (*n* = 3), radiographers (*n* = 3) and student radiographers (*n* = 14). However, only 10 members responded to the interview invite. The sample size for the study was therefore two nurses working in the maternity ward, two social workers, three radiographers and three student radiographers. Purposive sampling was employed to recruit participants as the study population was directly involved in the BoH intervention. The participants’ contact details, such as email addresses and telephone numbers, were therefore known to the researchers.

### Data collection method

The hospital-based participants within the interdisciplinary team, namely radiographers, nurses and social workers, were firstly called and informed about the nature of the study to be conducted. Secondly if they were interested in partaking, a follow-up email was then sent to them, which included a detailed information leaflet of the study and consent form. The volunteer student radiographers were also invited to partake in the study on their closed BoH intervention WhatsApp group. Students who were interested in partaking were requested to email the principal researcher. They were also provided with a follow-up email containing the information leaflet and consent form.

### Research instrument

Individual in-depth interviews were conducted telephonically or online. The online interviews were conducted on Microsoft Teams, and both online and telephonic interviews were audio recorded with the consent of the participants. The interviews were conducted over a period of 6 months from January to June 2022. The principal researcher conducted the interviews and employed reflexive bracketing to avoid bias. This was achieved by the researcher keeping a reflective journal to document any preconceived ideas about the participants’ experiences, both prior to the interview and during the analysis process. This was to make the researcher self-aware regarding the nature of probing questions, ensuring that her own views do not influence the participants. The interviews followed a semi-structured interview guide, which consisted of the following questions for all categories of the study population:

- *What were your overall experiences of the BoH intervention?*
- *Can you describe your experience of the interdisciplinary collaboration you encountered during the BoH intervention?*
- *What recommendations can you suggest towards ensuring the sustainability of the BoH intervention?*

The responses to these questions were followed up with probing questions to gain a more in-depth understanding of the impact the BoH intervention may have had on the stakeholders.

### Trustworthiness

Trustworthiness is an integral aspect of qualitative research to ensure scientific rigour (Enworo 2023). Maher et al. (2018) state that principles of credibility, dependability, confirmability and transferability must be applied to the research in order to assess trustworthiness (Maher et al. 2018). The individual interviews were transcribed verbatim, ensuring that the exact words of the participants were used and not summarised. The participants from all categories were asked the same neutral questions, such as ‘*what were your experiences regarding the BoH intervention*?’ This ensured that there were no leading questions and enabled the participants to share both positive and negative experiences. Dependability of the data collection and analysis process was ensured by creating a rich paper trail whereby all data from each category were correctly saved and analysed. Confirmability of the data analysis and the results was achieved by the co-researchers who were not involved in any aspect of the intervention and interviewing process. This enabled them to confirm the results without any bias from previous experiences. The study did not aim to generalise the findings; however, the detailed description of the intervention could support its application in other contexts, thus addressing the principle of transferability.

### Researcher’s characteristics

The research team consisted of the project coordinator and two external researchers not involved in the project to ensure that there is no conflict of interest in the interpretation of the results. The external researchers were from the radiography discipline and the Unit for Community Engagement. Their roles in the study, namely the data analysis, were critical towards ensuring unbiased and credible results.

### Data analysis

The audio recordings were transcribed and analysed by the researchers using reflective thematic analysis (RTA) described by Braun and Clark (Braun, Clarke & Hayfield 2022). This six-phase analytical process includes: (1) familiarisation with the data, (2) generating initial codes, (3) generating themes, (4) reviewing potential themes, (5) defining and naming the theme and (6) producing the report. In this study, the researchers applied the RTA steps by immersing themselves in the data and identifying common patterns that served as codes.

### Ethical considerations

The study gained ethical clearance from the University of Pretoria Research Ethics Committee (Reference No.: 90/2022) and the National Health Research Database. Permission was granted by the CEO of each hospital to participate in the intervention and to execute a research study from any data, provided ethical considerations were maintained. While this study did not directly engage with the beneficiaries of the BoH intervention, ethical principles were rigorously applied throughout the data collection and analysis process. These ethical principles included beneficence, self-determination, justice, confidentiality and non-maleficence.

The principle of beneficence was applied in this study by providing short-term support to abandoned babies and mothers until they regain stability. The beneficence of conducting this particular low-risk study was to ascertain how the BoH intervention could be sustained to ensure ongoing support to the community. Aspects related to sustainability in this context included the necessity and impact of the intervention, with the assumption that a positive impact would justify its continuation, guided by specific recommendations from the interdisciplinary team.

The principle of self-determination and justice was upheld whereby there was fairness in the selection process. In this study, all members of the interdisciplinary team were invited to partake in the interviews; however, only participants who responded to the invite were interviewed. The study also ensured that there was fair representation of all the sites where the BoH intervention was undertaken, by including health professionals from all hospitals.

The principle of confidentially was maintained by the researchers employing code names to each participant, ensuring that any personal identifiers such as names are omitted. Lastly, the principle of non-maleficence was ensured by not asking direct questions regarding scenarios where the participants may have interacted with abandoned babies, but rather allowing the participants to freely share what they felt comfortable with on their own accord. Participants were informed that if the views and experiences shared caused them discomfort, psychological support could be offered after the interview, through HospiVision, which is a hospital-based non-profit organisation (NPO) that offers counseling to both staff and students.

## Results

### Demographics

The study included 10 interdisciplinary members of the BoH team from various disciplines, namely nurses, social workers, radiographers and student radiographers. Their sociodemographic details, including designation and place of employment, are presented in Table 1.

**TABLE 1:** Sociodemographic details of the study population.

Category	Designation	Hospital
Nurse	Registered nurse	Hospital 1
	Registered nurse	Hospital 2
Social worker	Child welfare and family social worker	Hospital 1
	Child welfare and family social worker	Hospital 2
Radiographer	Chief radiographer	Hospital 1
	Chief radiographer	Hospital 2
	Senior radiographer	Hospital 3
Student radiographer	Final year	University of Pretoria
	Final year	University of Pretoria
	Final year	University of Pretoria

The generation of themes and subthemes from codes during the analytical process of this study is described in Table 2.

**TABLE 2:** Themes and sub-themes generated through the reflective thematic analysis process.

Themes	Sub-themes
1. The BoH intervention as a potential abandonment prevention strategy for hospital born infants	1.1 Reevaluation of the decision to abandon 1.2 Restored hope towards keeping the newborn baby
2. Psychological impact of the BoH intervention on the interdisciplinary team	2.1. Overwhelming experience 2.2. Coping mechanism for academic stress 2.3. Sensitisation towards patients’ socioeconomic challenges 2.4. Increased sense of gratitude and fulfilment
3. Strengthening of interdisciplinary collaboration and service delivery through the BoH intervention	3.1. Important sense of belonging among radiographers within the hospital team 3.2. Increased interprofessional collaboration among healthcare professionals 3.3. Enhanced service delivery among nurses
4. Sustainability strategies for the BoH intervention	4.1. Increased marketing and visibility of the BoH intervention 4.2. Executive management support

BoH, bags of hope.

Four themes were generated: Theme 1: The BoH intervention as a potential abandonment prevention strategy for hospital-born infants. Theme 2: Psychological impact of the BoH intervention on the interdisciplinary team. Theme 3: Strengthening of interdisciplinary collaboration through the BoH intervention. Theme 4: Sustainability strategies for the BoH intervention. The participant responses were coded as follows: social workers (SW), radiographers (R), student radiographers (SR) and nurses (N).

#### Theme 1: The bags of hope intervention as a potential abandonment prevention strategy for hospital-born infants

In the currently study, most of the beneficiaries of the BoH intervention included unemployed teenage mothers who had just given birth. Social workers in this intervention were primarily responsible for engaging with mothers of newborns to ascertain if they were in need of assistance. Based on these interactions, they reflected on how receiving a bag often changed the mothers’ views towards their situation. This was reported in the following narrations:

‘Shew, you have no idea. I wish I could video the expression on their faces. They are so grateful. When you hand over the bag and look at the mothers face you just want to cry. I had one patient who kneeled in front of me, she was so grateful. These bags are helping them have something. It is making such a positive impact on these mothers in very difficult socio-economic constraints. When we provide counselling and then give them the bag, it like gives them hope. Even if they wanted to abandon the baby, they think twice now, because they can see that at least they have some help. I had that happened to me, whereby it was a teenage mother and the boyfriend left her at 5 months, she then got another one, however he only wanted to support the mother and not the child. After counselling, when the mother left, she abandoned the baby outside the hospital and left. However, with the start of this intervention, that has never happened again.’ (SW1)

Another social worker shared the same views of the possibility of the bag providing hope to mothers in need, as indicated in the following narration:

‘I was having a conversation with the radiographer as she said we must fill in the form if there are any abandoned babies. I then said we don’t even have abandoned babies anymore because with this project, they are so happy and grateful to receive a bag that they keep the babies, even when they were considering to give them up after giving birth in hospital. So yes, this bag is acting as a preventative measure.’ (SW2)

From these narrations, it is evident that the BoH intervention may have brought temporary relief towards the mothers, as narrated by the participants. It is therefore assumed that the BoH intervention could serve as a prevention strategy towards infant abandonment within or outside of hospitals as described by the social worker, whereby the bags offer some relief to care for their newborn babies instead of having no provisions at all.

#### Theme 2: Psychological impact of the bags of hope intervention on the interdisciplinary team

Qualified and student radiographers indicated that the intervention had made a significant impact on their emotional state and thus their mental well-being. Radiographers described the enormity of positive feelings they experienced by saying:

‘It’s really touching. It’s such an overwhelming experience that we go through, and it makes you become full of emotions, seeing the impact that the bags of hope are doing to these kids.’ (R1)

A radiography student described how the intervention served as a distraction to academic-related stress, which was evident in the following participant’s response:

‘Especially with final year, it gets so hectic and overwhelming, but just doing this you literally forget it and seeing those beautiful babies you forget about anything. Even though I am struggling with academics. Babies have this innocence about them that make you feel like everything is going to be ok. It just made everything feel better.’ (SR3)

A student shared how the intervention heighted her awareness of the socioeconomic challenges patients face, recollecting an experience where she observed a newborn baby without any clothes. In addition, she noted how the intervention was able to restore dignity to the newborn baby. This was evident in the following narration:

‘A new born baby was wrapped in hospital blankets … they [*nurses*] were so grateful that the child would get, you know, warm clothes. The child would get at least a comfortable and decent blanket.’ (SR1)

Another student reflected on how the sensitisation to patient’s challenges made fostered a deeper sense of gratitude within herself through the following narration:

‘It really changed me in a way that I am grateful for the way I was brought up and that I have a loving mother and a father.’ (SR2)

Participant 3 further elaborated that the intervention enabled her to do something for someone else, which led to feelings of fulfilment.

‘You feel accomplished, you know, like doing something, knowing that you are not going to get anything in return. I feel like that feeling does not compare to anything. It’s just very satisfying and you feel so fulfilled.’ (SR3)

From these narrations it was evident that the BoH intervention evoked many positive emotions within the disciplinary team, which may have had a psychological impact on the team. This was particularly evident among student radiographers, who primarily interact with newborn babies during radiographic examinations and are often not aware of the socioeconomic backgrounds of the families they belong to.

#### Theme 3: Strengthening of interdisciplinary collaboration and service delivery through the bags of hope intervention

In this theme, a student radiographer explained how that the intervention made her realise the importance of interdisciplinary collaboration by saying:

‘I think it really made me feel how collaboration is really the body of the healthcare system. Like we working together hand in hand is how we meet needs. It’s not like we are isolated and just say I’m just relating with radiographers. So, when we get there, I get to actually interact with the other divisions. It really makes the whole. Then you see how the system needs every part of the healthcare professional.’ (SR1)

The social worker reflected how the BoH intervention has brought cohesion among the hospital staff and the radiography department in the following narration:

‘It has really changed the relationship. They are a part of us. We know that after screening the mother that if she is in need, we need to call [*radiographer*] and she will come with the student and the bags.’ (SW1)

‘Previously we as radiography as a department, we didn’t have much of the relationship with the social worker, but now they’re able to come to us. You know when they come to us, maybe we have a brief discussion about the case that they are requesting. So it was really improved which made us understand their work of duty.’ (R2)

From these narrations, it is evident that the BoH intervention played a significant role in connecting three disciplines, namely nurses, radiographers and social workers who do not often interact with each other. However, through the intervention and spirit of Ubuntu, they were able to work cohesively towards identifying beneficiaries in need and provisioning care packages. Not only did this assist the beneficiaries but also the nurses towards enhancing their care, as described in the following narrations:

‘The bags bring relief to us, in the bags there are wipes and nappies, we can now provide care, especially to our lodger babies [*babies waiting for a home, still residing in hospital*].’(N1)

‘They [*nurses*] are overwhelmed and they’re [*nurses*] trying to provide with what they have. So having these resources coming from the outside is so helpful.’ (SR1)

In this context, lodger babies are often infants who have been abandoned or whose mothers intend to place them for adoption. While hospitals provide medical care, they do not provide clothing or toiletries. Mothers facing dire socioeconomic challenges often lack these basic necessities, making it challenging for the nurses caring for the infants to meet their basic needs. The BoH intervention, however, bridges this gap by providing the infant with a care package containing essential care items that can be used for a few weeks. This assistance therefore ensures that nurses can continue with service delivery even if mothers do not come to hospital with the items needed.

#### Theme 4: Sustainability strategies for the Bags of Hope intervention

The rationale for conducting this study was to understand the impact of the intervention and methods of sustaining the intervention if it were found to be positive. Based on the previous themes, it was evident that the intervention had a positive impact on both the recipients and the interdisciplinary team. The participants then offered recommendations on how the project could be sustained in the long term.

Students, in particular, who were mostly involved in collecting and packaging the donations recommended increased marketing and visibility to the project. This was evident in the following narration:

‘To keep growing, I honestly think we just need to stay consistent and raise more awareness and raise we can about this to get more donations so that more babies can be helped through this project cause believe this is such a special project and it’s quite big and it really touches the lives of other people.’ (S3)

Radiographers further indicated that if management gets involved, staff will be aware that resources are available to assist the babies in need, as described in the following narration:

‘When there’s managers meetings, they always make an announcement that radiography has bags. If they are willing to announce it, some meetings at the end that managers do not forget, lets assess our child, let no child feel cold. We’ve got bags of hope in X Ray Department.’ (R2)

These recommendations towards sustainability of the intervention indicated that there is a need to continue to raise awareness about the intervention in order to attain donations from the public as well as to inform the health professionals of the project to extend its reach.

## Discussion

Child abandonment often stems from poverty and unwanted pregnancies, resulting in the mothers being unable to financially and emotionally support the child (Thabane & Kasiram 2015). The findings from the study indicate that the BoH intervention may have had an influence on the mother’s decision to keep the babies, given the decline of hospital-abandoned babies in these contexts following the commencement of the BoH intervention. Child abandonment strategies in developing countries such as Ghana include staff contributions to support care, donations from the public and strengthening of the hospital social welfare department (Abukari & Schmollgruber 2024). The findings from the current study are therefore in line with some of the child abandonment prevention strategies described by Abukari and Schmollgruber (2024), whereby donations from the public and institutions, such as the University of Pretoria, form the primary mode of assistance to babies in the earliest stages of abandonment who are placed in hospitals. Thabane and Kasiram (2015) support this approach, noting that providing mothers with access to resources could discourage them from abandoning their babies, as seen in Lesotho. In the United Kingdom, strategies towards infant abandonment remain limited. Mueller and Sherr state that there is much-needed basic incidence data in order to formulate risk factors and therefore inform abandonment prevention strategies (Meuller & Sherr 2009). The BoH intervention is presumed to contribute to preventing infant abandonment in this context, especially among mothers experiencing socioeconomic challenges. However, its impact is limited when addressing other factors linked to child abandonment, such as rape and gender-based violence, where the mother may not wish to keep the child. It is therefore recommended that incidence data be captured and analysed to broaden the support that can be offered.

The BoH intervention appeared to have a psychological impact on the members of the BoH interdisciplinary team. For radiographers, both qualified and students, participating in the intervention acclimatised them to the socioeconomic constraints that patients face. The act of providing care to vulnerable babies has allowed students to shift their focus from academic and professional stresses to positive, meaningful engagement. A study by Wu et al. identified an alternate coping strategy to support mental health (Wu, Styra & Gold 2020). This includes medical students offering prosocial charitable services such as carrying out errands for the elderly, delivering groceries and taking care of children (Wu et al. 2020). This assisted them in shifting the focus away from their stress into something positive (Wu et al. 2020). However, in this context, students are still within the clinical hospital training context servicing patients. This aligns well with the goal of service learning within higher education (Bringle & Hatcher 2009). The interdisciplinary team also described the overwhelming nature of the intervention. Similarly, caregivers of abandoned babies often experience intense emotions but may lack opportunities to debrief and process their feelings (Oosthuizen-Erasmus et al. 2022). However, unlike caregivers, health professionals offer temporary care to abandoned babies and have indicated that they gain a sense of fulfilment through the BoH intervention. The positive emotional feeling from helping others is termed the ‘Warm Glow Effect’, which has deep roots in philanthropy and psychology (Kurtz, Furnagiev & Forbes 2023). This phenomenon is the psychological effect on an individual when giving charity or performing acts of altruism, which result in private joy and prestige (Harbaugh 1995; Karns, Moore & Mayr 2017). It is therefore evident that intervention had a psychological impact on the team when providing the bags to the beneficiaries.

The intervention has significantly enhanced interdisciplinary collaboration among healthcare professionals towards a common goal of assisting vulnerable patients to whom they provide professional care (Rice 2000). Interdisciplinary collaboration in healthcare fosters a unified approach among professionals from diverse fields, enabling them to address complex patient needs more effectively. This approach has been shown to significantly improve teamwork and align efforts towards supporting vulnerable patients (Rice 2000). Villeneuve et al. (2020) state that it is impossible to ‘solve’ problems within a single discipline. Addressing social issues requires collaboration, combining and integrating different disciplines, perspectives and knowledge to identify and tackle the problem (Villeneuve et al. 2020). In this context, the BoH intervention, operating within community-recognised healthcare centres, may offer hope to mothers in need by providing accessible support. It is therefore essential for the intervention to identify strategies that ensure its sustainability.

Gebhardt, Brost and König (2019) elaborate for interventions to be sustainable; research undertaken must be highly interdisciplinary in nature to respond to the challenges surrounding the topic. Sustainability research should provide knowledge for action and is therefore strongly related with social and political issues such as regulation, behaviour, daily routines of users, consumption patterns, perceptions, attitudes and values (Gebhardt et al. 2019). To sustain the BoH intervention, several recommendations have been proposed by the participants. Choi et al. (2019) explain that organisations should establish a strong presence and awareness of their mission within their local community. This can lead to ongoing relationships with potential donors, thereby sustaining the intervention.

## Conclusion

The study describes experiences of the interdisciplinary team regarding the BoH intervention. While the intervention does not fully resolve the complex social issues associated with infant abandonment, it offers critical short-term support to help meet immediate needs until stability is regained through interdisciplinary collaboration. This approach has a symbiotic effect on both the recipients and the givers. The interdisciplinary team was psychologically impacted by the circumstances these babies faced and experienced the ‘warm glow’ effect when providing care items. The BoH intervention provides temporary support to vulnerable infants; however, for the intervention to have a lasting impact on the community, strategies for its sustainability need to be implemented. These strategies include raising public awareness of the intervention to garner more donations and increasing access to vulnerable infants by informing healthcare professionals about the project. Support from executive management can also play an important role in adopting and promoting this initiative. While these strategies can be used to address the sustainability of the current intervention, it is recommended that further research be done on the incidence data of hospital-abandoned babies to ascertain other factors leading to abandonment beyond financial instability. This could inform the involvement of other disciplines within the team to provide a broader holistic support. In conclusion, the study provides evidence that such interventions facilitated through higher education community engagement initiatives have the potential to bridge socioeconomic challenges and foster interdisciplinary collaboration.
